# Supplementary material for: The efficacy of topical, oral and surgical interventions for the treatment of tungiasis: A systematic review of the literature
Source: PLoS Negl Trop Dis. 2021 Aug 20;15(8):e0009722. doi: 10.1371/journal.pntd.0009722 (PMC8409605; doi:10.1371/journal.pntd.0009722)
Supplement: S1 PRISMA Checklist — (DOCX) [file pntd.0009722.s001.docx]

| **Section and Topic** | **Item #** | **Checklist item** | **Location where item is reported** |
| --- | --- | --- | --- |
| **TITLE** | | |  |
| Title | 1 | Identify the report as a systematic review. | Title |
| **ABSTRACT** | | |  |
| Abstract | 2 | See the PRISMA 2020 for Abstracts checklist. | Abstract |
| **INTRODUCTION** | | |  |
| Rationale | 3 | Describe the rationale for the review in the context of existing knowledge. | It is important to develop a clinical pathway for proper understanding of disease management (Figure 1). |
| Objectives | 4 | Provide an explicit statement of the objective(s) or question(s) the review addresses. | We aimed to perform a comprehensive systematic review of the literature to assess the efficacy of topical, oral and surgical interventions for the treatment of tungiasis. |
| **METHODS** | | |  |
| Eligibility criteria | 5 | Specify the inclusion and exclusion criteria for the review and how studies were grouped for the syntheses. | We included any type of clinical trial or longitudinal observational study that assessed patients diagnosed with tungiasis and that evaluated any topical treatment, systemic treatment or mechanical extraction method for parasite eradication. |
| Information sources | 6 | Specify all databases, registers, websites, organisations, reference lists and other sources searched or consulted to identify studies. Specify the date when each source was last searched or consulted. | Table 1  On September 1st, 2020, we comprehensively searched for articles in PubMed, EMBASE, Scopus, Web of Science, Science Direct, Scielo and LILACS BVS. |
| Search strategy | 7 | Present the full search strategies for all databases, registers and websites, including any filters and limits used. | Table 1 |
| Selection process | 8 | Specify the methods used to decide whether a study met the inclusion criteria of the review, including how many reviewers screened each record and each report retrieved, whether they worked independently, and if applicable, details of automation tools used in the process. | We included any type of clinical trial or longitudinal observational study that assessed patients diagnosed with tungiasis and that evaluated any topical treatment, systemic treatment or mechanical extraction method for parasite eradication. We excluded case series, case reports and trials that evaluated only environmental strategies for the control of tungiasis. All selected titles and abstracts were exported to EPPI-Reviewer 4 Version 4.6.4.0 (EPPI Centre, London, UK), and duplicates were removed. Two independent reviewers (CG & AM) screened the titles and abstracts and subsequently screened the selected full texts. Disagreements regarding title and abstract evaluation and full-text evaluation were resolved by a third independent reviewer (PK). |
| Data collection process | 9 | Specify the methods used to collect data from reports, including how many reviewers collected data from each report, whether they worked independently, any processes for obtaining or confirming data from study investigators, and if applicable, details of automation tools used in the process. | Data extraction was performed by two independent reviewers (CG & AM), and disagreements were resolved by consensus. |
| Data items | 10a | List and define all outcomes for which data were sought. Specify whether all results that were compatible with each outcome domain in each study were sought (e.g. for all measures, time points, analyses), and if not, the methods used to decide which results to collect. | The form collected important information about the publication year, the geographical area where the study was conducted, the number of patients included, the inclusion of indigenous communities, or children, the treatment of disseminated tungiasis, the presence of complications related to tungiasis, the type of treatment and treatment outcomes. |
|  | 10b | List and define all other variables for which data were sought (e.g. participant and intervention characteristics, funding sources). Describe any assumptions made about any missing or unclear information. | The form collected important information about the publication year, the geographical area where the study was conducted, the number of patients included, the inclusion of indigenous communities, or children, the treatment of disseminated tungiasis, the presence of complications related to tungiasis, the type of treatment and treatment outcomes. |
| Study risk of bias assessment | 11 | Specify the methods used to assess risk of bias in the included studies, including details of the tool(s) used, how many reviewers assessed each study and whether they worked independently, and if applicable, details of automation tools used in the process. | We evaluated the possible risk of bias (RoB) in clinical trials using the Revised Cochrane Risk of Bias Tool for Randomized Trials. For observational studies, we used the Critical Appraisal Tool from the Joanna Briggs Institute. Both analyses were performed by two independent reviewers (CG & AM), and disagreements were resolved by consensus. |
| Effect measures | 12 | Specify for each outcome the effect measure(s) (e.g. risk ratio, mean difference) used in the synthesis or presentation of results. | Considering that most articles presented great variability in outcome measures, the quantitative synthesis was based on frequencies and on individual relative risks. No additional quantitative synthesis method was performed due to the evident heterogeneity in article methods, intervention reporting, outcome measurement and quality analysis. |
| Synthesis methods | 13a | Describe the processes used to decide which studies were eligible for each synthesis (e.g. tabulating the study intervention characteristics and comparing against the planned groups for each synthesis (item #5)). | Considering that most articles presented great variability in outcome measures, the quantitative synthesis was based on frequencies and on individual relative risks. No additional quantitative synthesis method was performed due to the evident heterogeneity in article methods, intervention reporting, outcome measurement and quality analysis. |
|  | 13b | Describe any methods required to prepare the data for presentation or synthesis, such as handling of missing summary statistics, or data conversions. | Considering that most articles presented great variability in outcome measures, the quantitative synthesis was based on frequencies and on individual relative risks. No additional quantitative synthesis method was performed due to the evident heterogeneity in article methods, intervention reporting, outcome measurement and quality analysis. |
|  | 13c | Describe any methods used to tabulate or visually display results of individual studies and syntheses. | Considering that most articles presented great variability in outcome measures, the quantitative synthesis was based on frequencies and on individual relative risks. No additional quantitative synthesis method was performed due to the evident heterogeneity in article methods, intervention reporting, outcome measurement and quality analysis. |
|  | 13d | Describe any methods used to synthesize results and provide a rationale for the choice(s). If meta-analysis was performed, describe the model(s), method(s) to identify the presence and extent of statistical heterogeneity, and software package(s) used. | Considering that most articles presented great variability in outcome measures, the quantitative synthesis was based on frequencies and on individual relative risks. No additional quantitative synthesis method was performed due to the evident heterogeneity in article methods, intervention reporting, outcome measurement and quality analysis. |
|  | 13e | Describe any methods used to explore possible causes of heterogeneity among study results (e.g. subgroup analysis, meta-regression). | Considering that most articles presented great variability in outcome measures, the quantitative synthesis was based on frequencies and on individual relative risks. No additional quantitative synthesis method was performed due to the evident heterogeneity in article methods, intervention reporting, outcome measurement and quality analysis. |
|  | 13f | Describe any sensitivity analyses conducted to assess robustness of the synthesized results. | Considering that most articles presented great variability in outcome measures, the quantitative synthesis was based on frequencies and on individual relative risks. No additional quantitative synthesis method was performed due to the evident heterogeneity in article methods, intervention reporting, outcome measurement and quality analysis. |
| Reporting bias assessment | 14 | Describe any methods used to assess risk of bias due to missing results in a synthesis (arising from reporting biases). | We evaluated the possible risk of bias (RoB) in clinical trials using the Revised Cochrane Risk of Bias Tool for Randomized Trials. For observational studies, we used the Critical Appraisal Tool from the Joanna Briggs Institute. Both analyses were performed by two independent reviewers (CG & AM), and disagreements were resolved by consensus. |
| Certainty assessment | 15 | Describe any methods used to assess certainty (or confidence) in the body of evidence for an outcome. | We evaluated the possible risk of bias (RoB) in clinical trials using the Revised Cochrane Risk of Bias Tool for Randomized Trials. For observational studies, we used the Critical Appraisal Tool from the Joanna Briggs Institute. Both analyses were performed by two independent reviewers (CG & AM), and disagreements were resolved by consensus. |
| **RESULTS** | | |  |
| Study selection | 16a | Describe the results of the search and selection process, from the number of records identified in the search to the number of studies included in the review, ideally using a flow diagram. | Figure 2 |
|  | 16b | Cite studies that might appear to meet the inclusion criteria, but which were excluded, and explain why they were excluded. | Figure 2 |
| Study characteristics | 17 | Cite each included study and present its characteristics. | Our search strategy resulted in 3376 references. We excluded 808 duplicates and evaluated 2568 titles and abstracts. Subsequently, we screened 114 full texts (11 were not retrieved) and finally included 19 articles. Nine articles reported results of clinical trials for tungiasis treatments,[8,9,11–17] and ten reported the results of observational studies (Figure 2).[3,5,18–26] |
| Risk of bias in studies | 18 | Present assessments of risk of bias for each included study. | Figure 3, S3 Table |
| Results of individual studies | 19 | For all outcomes, present, for each study: (a) summary statistics for each group (where appropriate) and (b) an effect estimate and its precision (e.g. confidence/credible interval), ideally using structured tables or plots. | Tables 2 and 3 |
| Results of syntheses | 20a | For each synthesis, briefly summarise the characteristics and risk of bias among contributing studies. | Figure 3, S3 Table |
|  | 20b | Present results of all statistical syntheses conducted. If meta-analysis was done, present for each the summary estimate and its precision (e.g. confidence/credible interval) and measures of statistical heterogeneity. If comparing groups, describe the direction of the effect. | Tables 2 and 3 |
|  | 20c | Present results of all investigations of possible causes of heterogeneity among study results. | Tables 2 and 3 |
|  | 20d | Present results of all sensitivity analyses conducted to assess the robustness of the synthesized results. | Tables 2 and 3 |
| Reporting biases | 21 | Present assessments of risk of bias due to missing results (arising from reporting biases) for each synthesis assessed. | Figure 3, S3 Table |
| Certainty of evidence | 22 | Present assessments of certainty (or confidence) in the body of evidence for each outcome assessed. | Figure 3, S3 Table |
| **DISCUSSION** | | |  |
| Discussion | 23a | Provide a general interpretation of the results in the context of other evidence. | Although the final number of studies (n = 19) included in the review was not extremely low, our search strategy revealed that tungiasis has been neglected to a greater extent than diseases such as leishmaniasis and leprosy. |
|  | 23b | Discuss any limitations of the evidence included in the review. | Although a relatively low quantity of scientific studies is always a limitation for systematic reviews targeting the treatment of neglected diseases, we selected 5 studies that did not present a high RoB. Limitations related to high heterogeneity among comparisons and outcomes preclude any detailed quantitative synthesis and may jeopardize the evaluation of reproducibility of the results. |
|  | 23c | Discuss any limitations of the review processes used. | Although a relatively low quantity of scientific studies is always a limitation for systematic reviews targeting the treatment of neglected diseases, we selected 5 studies that did not present a high RoB. Limitations related to high heterogeneity among comparisons and outcomes preclude any detailed quantitative synthesis and may jeopardize the evaluation of reproducibility of the results. |
|  | 23d | Discuss implications of the results for practice, policy, and future research. | Although a relatively low quantity of scientific studies is always a limitation for systematic reviews targeting the treatment of neglected diseases, we selected 5 studies that did not present a high RoB. Limitations related to high heterogeneity among comparisons and outcomes preclude any detailed quantitative synthesis and may jeopardize the evaluation of reproducibility of the results. |
| **OTHER INFORMATION** | | |  |
| Registration and protocol | 24a | Provide registration information for the review, including register name and registration number, or state that the review was not registered. | We strictly followed a predesigned review protocol registered in PROSPERO (CRD42021234741). |
|  | 24b | Indicate where the review protocol can be accessed, or state that a protocol was not prepared. | We strictly followed a predesigned review protocol registered in PROSPERO (CRD42021234741). |
|  | 24c | Describe and explain any amendments to information provided at registration or in the protocol. | We strictly followed a predesigned review protocol registered in PROSPERO (CRD42021234741). |
| Support | 25 | Describe sources of financial or non-financial support for the review, and the role of the funders or sponsors in the review. | This work was supported by Conselho Nacional de Desenvolvimento Científico e Tecnológico e Ministério da Saúde, MS-SCTIE-Decit No. 22/2019, Brazil. |
| Competing interests | 26 | Declare any competing interests of review authors. | Conflicts of interest  None. |
| Availability of data, code and other materials | 27 | Report which of the following are publicly available and where they can be found: template data collection forms; data extracted from included studies; data used for all analyses; analytic code; any other materials used in the review. | S1 Table. Table containing the complete data extracted in the review process. |

*From:*  Page MJ, McKenzie JE, Bossuyt PM, Boutron I, Hoffmann TC, Mulrow CD, et al. The PRISMA 2020 statement: an updated guideline for reporting systematic reviews. BMJ 2021;372:n71. doi: 10.1136/bmj.n71

For more information, visit: <http://www.prisma-statement.org/>
