# Supplementary material for: The efficacy of topical, oral and surgical interventions for the treatment of tungiasis: A systematic review of the literature
Source: PLoS Negl Trop Dis. 2021 Aug 20;15(8):e0009722. doi: 10.1371/journal.pntd.0009722 (PMC8409605; doi:10.1371/journal.pntd.0009722)
Supplement: S1 File — (DOCX) [file pntd.0009722.s002.docx]

The efficacy of oral and topical interventions in comparison to surgical extraction for the treatment for tungiasis: a systematic review of the literature.

**Thesis databases searched**

1) Queen’s Univerity Library Catalogue

<https://ocul-qu.primo.exlibrisgroup.com/discovery/fulldisplay?docid=proquest1832307937&context=PC&vid=01OCUL_QU:QU_DEFAULT&lang=en&search_scope=MyInst_and_CI&adaptor=Primo%20Central&tab=Everything&query=any,contains,resistence%20M.%20leprae&facet=rtype,exclude,reviews&facet=rtype,exclude,articles&facet=rtype,exclude,other&offset=0>

<https://ocul-qu.primo.exlibrisgroup.com/discovery/fulldisplay?docid=pubmed_central6334779&context=PC&vid=01OCUL_QU:QU_DEFAULT&lang=en&search_scope=MyInst_and_CI&adaptor=Primo%20Central&tab=Everything&query=any,contains,resistence%20M.%20leprae&facet=rtype,exclude,reviews&facet=rtype,exclude,articles&facet=rtype,exclude,other&offset=0>

<https://ocul-qu.primo.exlibrisgroup.com/discovery/fulldisplay?docid=proquest250907108&context=PC&vid=01OCUL_QU:QU_DEFAULT&lang=en&search_scope=MyInst_and_CI&adaptor=Primo%20Central&tab=Everything&query=any,contains,resistence%20M.%20leprae&facet=rtype,exclude,reviews&facet=rtype,exclude,articles&facet=rtype,exclude,other&offset=0>

2) Queen’s Univerity Research & Learning Repository (QSpace): Theses & Dissertations

<https://qspace.library.queensu.ca/discover?scope=%2F&query=leprosy+&submit=>

<https://qspace.library.queensu.ca/discover?scope=%2F&query=leprosy+and+resist*&submit=>

3) Thesis Canada Portal

<https://www.bac-lac.gc.ca/eng/services/theses/Pages/list.aspx?AW_S=leprosy>

<https://www.bac-lac.gc.ca/eng/Search/Pages/results.aspx?k=,leprosy%20and%20resist*>

4) The Directory of Open Access Repositories

<http://v2.sherpa.ac.uk/cgi/search/repository/basic?repository_name-auto=leprosy&_action_search=Search&screen=Search&repository_name-auto_merge=ALL>

<http://v2.sherpa.ac.uk/cgi/search/repository/basic?repository_name-auto=leprosy+and+resist*&_action_search=Search&screen=Search&repository_name-auto_merge=ALL>

5) ProQuest Open Access Dissertations and Thesis

<https://pqdtopen.proquest.com/results.html?QryTxt=leprosy&fromyear=&toyear=&author=&Title=&pubnum=&school=&advisor=&keywords=>

<https://pqdtopen.proquest.com/results.html?QryTxt=leprosy+and+resist*&fromyear=&toyear=&author=&Title=&pubnum=&school=&advisor=&keywords=>

6) OpenThesis.org

<http://www.openthesis.org/search/search.html?from=advancedSearch&queryString=leprosy&searchButton.x=77&searchButton.y=22&sortOrder=Relevance&fromDate=&toDate=>

<http://www.openthesis.org/search/search.html?from=advancedSearch&queryString=leprosy+and+resist*&searchButton.x=58&searchButton.y=26&sortOrder=Relevance&fromDate=&toDate=>

7) Center for Research Libraries

<http://catalog.crl.edu/search/Y?SEARCH=leprosy&searchscope=4&x=6&y=15>

<http://catalog.crl.edu/search~S4/?searchtype=Y&searcharg=leprosy+and+resist*&searchscope=4&sortdropdown=-&SORT=DZ&extended=0&SUBMIT=Search&searchlimits=&searchorigarg=Yleprosy>

8) Networked Digital Library of Theses and Dissertations

<http://search.ndltd.org/show.php?id=oai%3Aunion.ndltd.org%3AIBICT%2Foai%3Ahttp%3A%2F%2Flocalhost%3Atede%2F5195&back=http%3A%2F%2Fsearch.ndltd.org%2Fsearch.php%3Fq%3DMycobacterium%2Bleprae%2Bresistence>

<http://search.ndltd.org/show.php?id=oai%3Aunion.ndltd.org%3AIBICT%2Foai%3Arepositorio.unesp.br%3A11449%2F126493&back=http%3A%2F%2Fsearch.ndltd.org%2Fsearch.php%3Fq%3DMycobacterium%2Bleprae%2Bresistence%26start%3D70>

9) National Library of Australia’s Trove Service

<https://trove.nla.gov.au/book/result?l-format=Thesis&q=leprosy>

<https://trove.nla.gov.au/book/result?l-format=Thesis&q=leprosy+and+resist*>

10) United Kingdom E-Theses Online Service

<https://ethos.bl.uk/SearchResults.do>

11) Système universitaire de documentation

[http://www.sudoc.abes.fr/cbs/xslt//DB=2.1/SET=1/TTL=1/CMD?ACT=SRCHA&IKT=1016&SRT=RLV&TRM=leprosy](http://www.sudoc.abes.fr/cbs/xslt/DB=2.1/SET=1/TTL=1/CMD?ACT=SRCHA&IKT=1016&SRT=RLV&TRM=leprosy)

[http://www.sudoc.abes.fr/cbs/xslt//DB=2.1/SET=3/TTL=41/CMD?ACT=SRCHA&IKT=1016&SRT=RLV&TRM=leprosy+and+resist*](http://www.sudoc.abes.fr/cbs/xslt/DB=2.1/SET=3/TTL=41/CMD?ACT=SRCHA&IKT=1016&SRT=RLV&TRM=leprosy+and+resist*)

12) Fichier central des thèses

<http://theses.fr/fr/?q=leprosy+>

<http://theses.fr/fr/?q=leprosy+and+resist*>

13) Deutschen National Bibliothek

<https://portal.dnb.de/opac.htm;jsessionid=owdIe0sEHKktJeCfjgJeI114LfKZAp0V9OkzUmAK.prod-fly8?query=leprosy&method=simpleSearch>

<https://portal.dnb.de/opac.htm;jsessionid=owdIe0sEHKktJeCfjgJeI114LfKZAp0V9OkzUmAK.prod-fly8?query=leprosy+and+resist*&method=simpleSearch>

14) Tesis del Sistema Bibliotecario de la Universidad Nacional Autónoma de México

<http://oreon.dgbiblio.unam.mx/F/72ENSHK7MABEI5HDMEI3KIIUDQG7JQPXC7J2IS2NNNXXA8JHMN-09139?func=find-b&request=leprosy&find_code=WRD&adjacent=N&local_base=TES01&x=73&y=14&filter_code_2=WYR&filter_request_2=&filter_code_3=WYR&filter_request_3=>

<http://oreon.dgbiblio.unam.mx/F/72ENSHK7MABEI5HDMEI3KIIUDQG7JQPXC7J2IS2NNNXXA8JHMN-15163?func=find-b&REQUEST=leprosy+and+resist*&find_code=WRD&ADJACENT=N&local_base=TES01&x=41&y=16&filter_code_2=WYR&filter_request_2=&filter_code_3=WYR&filter_request_3=>

15) National Academic Research and Collaborations Information System

<https://www.narcis.nl/search/uquery/leprosy/Language/EN>

<https://www.narcis.nl/search/coll/research/uquery/leprosy%20and%20resist*/Language/EN>

16) Spain’s Ministry of Education thesis database

<https://educacion.gob.es/teseo/irGestionarConsulta.do;jsessionid=8E95C59D40FB1494AD4171D822F9CB93>

17) Biblioteca Virtual Miguel de Cervantes

<http://www.cervantesvirtual.com/buscador/?q=leprosy>

<http://www.cervantesvirtual.com/buscador/?q=leprosy+and+resist*>

18) Universidad Complutense de Madrid’s catalogue

<https://ucm.on.worldcat.org/search?databaseList=&queryString=leprosy>

<https://ucm.on.worldcat.org/search?sortKey=LIBRARY&databaseList=1953%2C1941%2C2259%2C2237%2C2269%2C3860%2C1672%2C3036%2C638%2C3954%2C3867&queryString=leprosy+and+resist*&changedFacet=&overrideStickyFacetDefault=&stickyFacetsChecked=on&selectSortKey=LIBRARY&clusterResults=off&scope=sz%3A37703&scope=sz%3A37628&subformat=Book%3A%3Abook_thsis&year=all&yearFrom=&yearTo=&author=all&language=all&database=all>

19) Tesis Doctorals en Xarxa

<https://tdx.cat/discover?scope=%2F&query=leprosy&submit=>

<https://tdx.cat/discover?scope=%2F&query=leprosy+resist*&submit=&scope=%2F>

20) Swiss National Library

<https://www.nb.admin.ch/snl/de/home/suche.html#leprosy>

<https://www.nb.admin.ch/snl/de/home/suche.html#leprosy%20resist*>

21) Universidade de São Paulo

<https://teses.usp.br/index.php?option=com_jumi&fileid=12&Itemid=77&lang=pt-br&filtro=leprosy>

<https://teses.usp.br/index.php?option=com_jumi&fileid=11&Itemid=76&lang=pt-br&filtro=leprosy%20resist*>

22) Universidade Federal de São Paulo

<https://repositorio.unifesp.br/discover?query=leprosy&filtertype=type&filter_relational_operator=equals&filter=Tese+de+doutorado>

<https://repositorio.unifesp.br/discover?scope=%2F&query=leprosy+resist*&submit=Ir&filtertype_0=type&filter_0=Tese+de+doutorado&filter_relational_operator_0=equals&rpp=10>

23) Universidade Estadual de Campinas

<http://www.bibliotecadigital.unicamp.br/document/list_index.php?palavra=leprosy&type_list=3&tid=498&stid%5B%5D=0&sent=s>

<http://www.bibliotecadigital.unicamp.br/document/list_index.php?palavra=leprosy+resist*&type_list=3&tid=498&sent=s>

24) Universidade Estadual Paulista

<https://repositorio.unesp.br/handle/11449/77291/discover?query=leprosy+&submit=>

<https://repositorio.unesp.br/handle/11449/77291/discover?query=leprosy+resist*&submit=>

25) Pontifícia Universidade Católica de Campinas

<http://www.bibliotecadigital.puc-campinas.edu.br/tde_busca/processaPesquisa.php?nrPagina=1&pesqExecutada=0&nrExpressoes=1&campo%5B0%5D=TODOS&texto%5B0%5D=leprosy+&Submit=Buscar+%BB&qtdRegPagina=5>

<http://www.bibliotecadigital.puc-campinas.edu.br/tde_busca/processaPesquisa.php?nrPagina=1&pesqExecutada=0&nrExpressoes=1&campo%5B0%5D=TODOS&texto%5B0%5D=leprosy+resist*&Submit=Buscar+%BB&qtdRegPagina=5>

26) Universidade Católica de Santos

<http://biblioteca.unisantos.br:8181/simple-search?query=leprosy>

<http://biblioteca.unisantos.br:8181/simple-search?location=%2F&query=leprosy+resist*&rpp=10&sort_by=score&order=desc>

27) Universidade de Taubaté

<https://unitau.br/busca/?termo=leprosy>

<https://unitau.br/busca/?termo=leprosy+and+resist*>

28) Universidade do Oeste Paulista

<http://bdtd.unoeste.br:8080/jspui/simple-search?query=leprosy>

<http://bdtd.unoeste.br:8080/jspui/simple-search?location=%2F&query=leprosy+resist*&rpp=10&sort_by=score&order=desc>

29) Universidade Presbiteriana Mackenzie

<http://tede.mackenzie.br/jspui/simple-search?query=leprosy>

<http://tede.mackenzie.br/jspui/simple-search?location=%2F&query=leprosy+resist*&rpp=10&sort_by=score&order=desc>

30) Universidade Metodista de São Paulo

<http://portal.metodista.br/posreligiao/publicacoes/teses-e-dissertacoes/teses-de-doutorado-1993-a-2003-1>

<http://portal.metodista.br/posreligiao/publicacoes/teses-e-dissertacoes/teses-de-doutorado-2004-a-2009>

<http://portal.metodista.br/posreligiao/publicacoes/teses-e-dissertacoes/teses-de-doutorado-2010-a-2019>

<http://portal.metodista.br/posreligiao/publicacoes/teses-e-dissertacoes/dissertacoes-de-mestrado-1981-a-2000-1>

<http://portal.metodista.br/posreligiao/publicacoes/teses-e-dissertacoes/dissertacoes-de-mestrado-2001-a-2009-1>

<http://portal.metodista.br/posreligiao/publicacoes/teses-e-dissertacoes/dissertacoes-de-mestrado-2010-a-2019>

31) Universidade Anhembi Morumbi

<http://sitios.anhembi.br/tedesimplificado/browse?type=department&sort_by=1&order=ASC&rpp=20&etal=-1&authority=3930158860712739524&starts_with=leprosy>

<http://sitios.anhembi.br/tedesimplificado/browse?type=department&sort_by=1&order=ASC&rpp=20&etal=-1&authority=3930158860712739524&starts_with=leprosy+resist*>

32) Universidade Nove de Julho

<https://bibliotecatede.uninove.br/simple-search?query=leprosy>

<https://bibliotecatede.uninove.br/simple-search?location=%2F&query=leprosy+resist*&rpp=10&sort_by=score&order=desc>

33) Universidade Federal de São Carlos

<https://repositorio.ufscar.br/handle/ufscar/1538/discover?query=leprosy+&submit=> <https://repositorio.ufscar.br/handle/ufscar/1538/discover?query=leprosy+resist*&submit=>

34) Universidade Federal do Acre

<http://www2.ufac.br/ppge/@@busca?SearchableText=leprosy>

<http://www2.ufac.br/ppge/@@busca?SearchableText=leprosy+resist*>

35) Universidade Federal de Alagoas

<http://www.repositorio.ufal.br/simple-search?query=leprosy>

<http://www.repositorio.ufal.br/simple-search?location=%2F&query=leprosy+resist*&rpp=10&sort_by=score&order=desc>

36) Universidade Federal do Amazonas

<https://tede.ufam.edu.br/simple-search?query=leprosy>

<https://tede.ufam.edu.br/simple-search?location=%2F&query=leprosy+resist*&rpp=10&sort_by=score&order=desc>

37) Universidade Estadual de Feira de Santana

<http://tede2.uefs.br:8080/simple-search?query=leprosy>

<http://tede2.uefs.br:8080/simple-search?location=%2F&query=leprosy+resist*&rpp=10&sort_by=score&order=desc>

38) Universidade de Federal da Bahia

<https://repositorio.ufba.br/ri/simple-search?query=leprosy&submit=Ir>

<https://repositorio.ufba.br/ri/simple-search?location=%2F&query=leprosy+resist*&rpp=10&sort_by=score&order=desc>

39) Universidade do Estado da Bahia

<http://www.cdi.uneb.br/site/?cat-trabalhos-academicos=pgdr&termo=filtro-todos&termo-valor=leprosy>

<http://www.cdi.uneb.br/site/?cat-trabalhos-academicos=pgdr&termo=filtro-todos&termo-valor=leprosy+resist*>

40) Universidade Federal do Ceará

<http://www.repositorio.ufc.br/simple-search?query=leprosy>

<http://www.repositorio.ufc.br/simple-search?location=%2F&query=leprosy+resist*&rpp=10&sort_by=score&order=desc>

41) Universidade de Brasília

<https://repositorio.unb.br/simple-search?query=leprosy>

<https://repositorio.unb.br/simple-search?location=%2F&query=leprosy+resist*&rpp=10&sort_by=score&order=desc>

42) Universidade Católica de Brasília

<https://bdtd.ucb.br:8443/jspui/simple-search?query=leprosy>

<https://bdtd.ucb.br:8443/jspui/simple-search?location=%2F&query=leprosy+resist*&rpp=10&sort_by=score&order=desc>

43) Universidade Federal do Espírito Santo

<http://repositorio.ufes.br/simple-search?query=leprosy>

<http://repositorio.ufes.br/simple-search?location=%2F&query=leprosy+resist*&rpp=10&sort_by=score&order=desc>

44) Universidade Federal de Goiás

<https://repositorio.bc.ufg.br/tede/simple-search?query=leprosy>

<https://repositorio.bc.ufg.br/tede/simple-search?location=%2F&query=leprosy+resist*&rpp=10&sort_by=score&order=desc>

45) Universidade Católica de Goiás

<http://tede2.pucgoias.edu.br:8080/simple-search?query=leprosy>

<http://tede2.pucgoias.edu.br:8080/simple-search?location=%2F&query=leprosy+resist*&rpp=10&sort_by=score&order=desc>

46) Universidade Federal Mato Grosso do Sul

<https://repositorio.ufms.br:8443/jspui/handle/123456789/52/browse?type=subject&order=ASC&rpp=20&starts_with=leprosy>

<https://repositorio.ufms.br:8443/jspui/handle/123456789/52/browse?type=subject&order=ASC&rpp=20&starts_with=leprosy+resist*>

47) Universidade Federal da Grande Dourados

<http://repositorio.ufgd.edu.br/jspui/simple-search?query=leprosy>

<http://repositorio.ufgd.edu.br/jspui/simple-search?location=%2F&query=leprosy+resist*&rpp=10&sort_by=score&order=desc>

48) Universidade Católica Dom Bosco

<https://site.ucdb.br/cursos/4/mestrado-e-doutorado/32/mestrado-e-doutorado-em-educacao/13167/doutorado-em-educacao/13192/teses-defendidas/13197/#busca=leprosy>

<https://site.ucdb.br/cursos/4/mestrado-e-doutorado/32/mestrado-e-doutorado-em-educacao/13167/doutorado-em-educacao/13192/teses-defendidas/13197/#busca=leprosy%20and%20resist*>

49) Universidade Federal de Uberlândia

<http://repositorio.ufu.br/simple-search?query=leprosy>

<http://repositorio.ufu.br/simple-search?location=%2F&query=leprosy+resist*&rpp=10&sort_by=score&order=desc>

50) Universidade Federal de Juiz de Fora

<http://repositorio.ufjf.br:8080/jspui/simple-search?query=leprosy>

<http://repositorio.ufjf.br:8080/jspui/simple-search?location=%2F&query=leprosy+resist*&rpp=10&sort_by=score&order=desc>

51) Universidade Federal de Lavras

<http://repositorio.ufla.br/simple-search?query=leprosy+>

<http://repositorio.ufla.br/simple-search?location=%2F&query=leprosy+and+resist*&rpp=10&sort_by=score&order=desc>

52) Universidade Federal de Minas Gerais

<https://repositorio.ufmg.br/simple-search?query=leprosy>

<https://repositorio.ufmg.br/simple-search?location=%2F&query=leprosy+resist*&rpp=10&sort_by=score&order=desc>

53) Universidade Federal de Ouro Preto

<https://www.repositorio.ufop.br/handle/123456789/8681/browse?type=subject&order=ASC&rpp=20&starts_with=hansen%C3%ADase>

54) Universidade Federal de Viçosa

<https://www.locus.ufv.br/handle/123456789/1/discover?query=leprosy+&submit=Ir>

<https://www.locus.ufv.br/handle/123456789/1/discover?query=leprosy+resist*&submit=Ir>

55) Pontifícia Universidade Católica de Minas Gerais

<https://web.sistemas.pucminas.br/BDP/PUC%20Minas>

56) Centro Universitário de Caratinga

<http://bibliotecadigital.unec.edu.br/bdtdunec/tde_busca/processaPesquisa.php?PHPSESSID=acfa08ae6d1b191ae595392d2092f8fa&nrPagina=1&pesqExecutada=0&nrExpressoes=1&campo%5B0%5D=TODOS&texto%5B0%5D=leprosy&Submit=Buscar+%BB&qtdRegPagina=5>

<http://bibliotecadigital.unec.edu.br/bdtdunec/tde_busca/processaPesquisa.php?nrPagina=1&pesqExecutada=0&nrExpressoes=1&campo%5B0%5D=TODOS&texto%5B0%5D=leprosy+resist*&Submit=Buscar+%BB&qtdRegPagina=5>

57) Universidade Federal do Pará

<http://www.repositorio.ufpa.br/jspui/handle/2011/2289/browse?type=subject&order=ASC&rpp=20&starts_with=leprosy>

<http://www.repositorio.ufpa.br/jspui/handle/2011/2289/browse?type=subject&order=ASC&rpp=20&starts_with=hansen%C3%ADase>

58) Universidade Federal da Paraíba

<https://repositorio.ufpb.br/jspui/simple-search?query=leprosy>

<https://repositorio.ufpb.br/jspui/simple-search?location=%2F&query=leprosy+resist*&rpp=10&sort_by=score&order=desc>

59) Universidade Federal do Paraná

<https://acervodigital.ufpr.br/handle/1884/284/discover?query=leprosy+&submit=>

<https://acervodigital.ufpr.br/handle/1884/284/discover?query=leprosy+resist*&submit=>

60) Pontifícia Universidade Católica do Paraná

<http://www.biblioteca.pucpr.br/tede/tde_busca/processaPesquisa.php?nrPagina=1&pesqExecutada=0&nrExpressoes=1&campo%5B0%5D=TODOS&texto%5B0%5D=leprosy&Submit=Buscar+%BB&qtdRegPagina=5>

<http://www.biblioteca.pucpr.br/tede/tde_busca/processaPesquisa.php?nrPagina=1&pesqExecutada=0&nrExpressoes=1&campo%5B0%5D=TODOS&texto%5B0%5D=leprosy+resist*&Submit=Buscar+%BB&qtdRegPagina=5>

61) Universidade Estadual de Londrina

<http://www.bibliotecadigital.uel.br/document/results.php?words=leprosy>

<http://www.bibliotecadigital.uel.br/document/results.php?method=and&sort=score&matchesperpage=10&words=leprosy+resist*>

62) Universidade Estadual do Oeste do Paraná

<http://tede.unioeste.br/simple-search?query=leprosy>

<http://tede.unioeste.br/simple-search?location=%2F&query=leprosy+resist*&rpp=10&sort_by=score&order=desc>

63) Universidade Estadual de Ponta Grossa

<https://tede2.uepg.br/jspui/handle/prefix/1/browse?type=subject&order=ASC&rpp=20&value=Leprosy>

64) Universidade Estadual de Maringá

<http://nou-rau.uem.br/nou-rau/document/results.php?words=leprosy>

<http://nou-rau.uem.br/nou-rau/document/results.php?words=leprosy+resist*>

65) Universidade Federal de Pernambuco

<https://repositorio.ufpe.br/handle/123456789/50/browse?type=subject&order=ASC&rpp=20&value=Leprosy>

66) Universidade de Pernambuco

<http://w2.portais.atrio.scire.net.br/upe-csaude/index.php/pt/doutorado/teses-doutorado>

67) Universidade Católica de Pernambuco

<http://tede2.unicap.br:8080/simple-search?query=leprosy>

68) Universidade Federal do Piauí

<https://repositorio.ufpi.br/xmlui/search?query=leprosy&submit=Ir>

<https://repositorio.ufpi.br/xmlui/search?scope=%2F&query=leprosy+resist*&rpp=10&sort_by=0&order=DESC&submit=Ir>

69) Universidade do Estado do Rio de Janeiro

<http://www.bdtd.uerj.br/tde_busca/processaPesquisa.php?nrPagina=1&pesqExecutada=0&nrExpressoes=1&campo%5B0%5D=TODOS&texto%5B0%5D=leprosy&Submit=Pesquisar+%BB&qtdRegPagina=5>

<http://www.bdtd.uerj.br/tde_busca/processaPesquisa.php?nrPagina=1&pesqExecutada=0&nrExpressoes=1&campo%5B0%5D=TODOS&texto%5B0%5D=leprosy+resist*&Submit=Pesquisar+%BB&qtdRegPagina=5>

70) Universidade Federal do Estado do Rio de Janeiro

<http://www2.unirio.br/unirio/ccbs/ppgenfbio/teses-1/teses-2018>

<http://www2.unirio.br/unirio/ccbs/ppgenfbio/teses-1/teses-ppgenfbio-unirio-ano-2017>

<http://www2.unirio.br/unirio/ccbs/ppgenfbio/teses-1/teses-ppgenfbio-unirio-ano-2016>

<http://www2.unirio.br/unirio/ccbs/ppgenfbio/teses-1/teses-ppgenfbio-unirio-ano-2015>

<http://www2.unirio.br/unirio/ccbs/ppgenfbio/teses-1/teses-ppgenfbio-unirio-2014>

<http://www2.unirio.br/unirio/ccbs/ppgenfbio/teses-1/teses-ppgenfbio-unirio-ano-2013>

71) Universidade Federal Fluminense

<https://app.uff.br/riuff/simple-search?query=leprosy>

<https://app.uff.br/riuff/simple-search?location=%2F&query=leprosy+resist*&rpp=10&sort_by=score&order=desc>

72) Universidade Federal do Rio de Janeiro

<https://pantheon.ufrj.br/handle/11422/1/browse?type=subject&order=ASC&rpp=20&value=Hanseniase>

<https://pantheon.ufrj.br/handle/11422/1/browse?type=subject&order=ASC&rpp=20&value=Hansen%C3%ADase>

73) Universidade Federal Rural do Rio de Janeiro

<https://tede.ufrrj.br/jspui/simple-search?query=leprosy>

74) Pontifícia Universidade Católica do Rio de Janeiro

<https://www.maxwell.vrac.puc-rio.br/colecao.php?strSearch=leprosy&strTit=&strAut=>

75) Fundação Oswaldo Cruz

<http://teses.icict.fiocruz.br/cgi-bin/wxis1660.exe/lildbi/iah/>

76) Universidade Federal do Rio Grande do Norte

<http://repositorio.ufrn.br:8080/jspui/simple-search?location=%2F&query=leprosy&rpp=10&sort_by=score&order=desc>

<http://repositorio.ufrn.br:8080/jspui/simple-search?query=leprosy+resist*&sort_by=score&order=desc&rpp=10&etal=0&start=10>

77) Universidade Potiguar

<http://eds.b.ebscohost.com/eds/results?vid=0&sid=a76f9ad1-abdb-4265-a305-22aba7e6f2d0%40pdc-v-sessmgr03&bquery=leprosy&bdata=Jmxhbmc9cHQtYnImdHlwZT0wJnNlYXJjaE1vZGU9QW5kJnNpdGU9ZWRzLWxpdmU%3d>

<http://eds.b.ebscohost.com/eds/results?vid=1&sid=a76f9ad1-abdb-4265-a305-22aba7e6f2d0%40pdc-v-sessmgr03&bquery=leprosy+and+resist*&bdata=Jmxhbmc9cHQtYnImdHlwZT0wJnNlYXJjaE1vZGU9QW5kJnNpdGU9ZWRzLWxpdmU%3d>

78) Universidade Federal do Rio Grande do Sul

<https://sabi.ufrgs.br/F/XHY991GN9UH98297D9UI6VX1YUXJ746348FNIN2DMY1XU18HKE-17446?func=find-b&request=leprosy&find_code=WRD&adjacent=N&x=49&y=5&filter_code_2=WLN&filter_request_2=&filter_code_3=WYR&filter_request_3=&filter_code_4=WYR&filter_request_4=>

<https://sabi.ufrgs.br/F/XHY991GN9UH98297D9UI6VX1YUXJ746348FNIN2DMY1XU18HKE-18539?func=find-b&request=leprosy+resist*&find_code=WRD&adjacent=N&x=35&y=11&filter_code_2=WLN&filter_request_2=&filter_code_3=WYR&filter_request_3=&filter_code_4=WYR&filter_request_4=>

79) Universidade Federal de Santa Maria

<https://repositorio.ufsm.br/discover?scope=%2F&query=leprosy+&submit=>

<https://repositorio.ufsm.br/discover?scope=%2F&query=leprosy+resist*&submit=>

80) Fundação Universidade Federal do Rio Grande

<https://lume.ufrgs.br/handle/10183/1/discover?query=leprosy&querytype_0=title&query_relational_operator_0=contains&query_value_0=&querytype_1=authortd&query_relational_operator_1=contains&query_value_1=&querytype_2=orientador&query_relational_operator_2=contains&query_value_2=&querytype_3=subject&query_relational_operator_3=contains&query_value_3=&querytype_5=dataAno&query_relational_operator_5=equals&query_value_5=&querytype_6=nivelAcademico&query_relational_operator_6=equals&query_value_6=&querytype_7=tipo&query_relational_operator_7=equals&query_value_7=&querytype_8=idioma&query_relational_operator_8=equals&query_value_8=&querytype_9=formatoArquivo&query_relational_operator_9=equals&query_value_9=&submit-search=&opened_filter=yes&mode=query&dateMode=select>

[https://lume.ufrgs.br/handle/10183/1/discover?rpp=10&etal=0&query=leprosy+resist*&group_by=none&page=2&querytype_0=title&query_relational_operator_0=contains&query_value_0=&querytype_1=authortd&query_relational_operator_1=contains&query_value_1=&querytype_10=serie&query_relational_operator_10=contains&query_value_10=&querytype_11=author&query_relational_operator_11=contains&query_value_11=&querytype_12=acervo&query_relational_operator_12=contains&query_value_12=&querytype_13=descriptionSection&query_relational_operator_13=contains&query_value_13=&querytype_14=tipoAto&query_relational_operator_14=contains&query_value_14=&querytype_15=natureza&query_relational_operator_15=contains&query_value_15=&querytype_16=numeroAto&query_relational_operator_16=contains&query_value_16=&querytype_17=orgao&query_relational_operator_17=contains&query_value_17=&querytype_18=dataFinal&query_relational_operator_18=contains&query_value_18=&querytype_19=programa&query_relational_operator_19=contains&query_value_19=&querytype_2=orientador&query_relational_operator_2=contains&query_value_2=&querytype_20=entrevistado&query_relational_operator_20=contains&query_value_20=&querytype_21=grandeArea&query_relational_operator_21=contains&query_value_21=&querytype_22=tipoDeApresentacao&query_relational_operator_22=contains&query_value_22=&querytype_23=areaTematica&query_relational_operator_23=contains&query_value_23=&querytype_24=coordenador&query_relational_operator_24=contains&query_value_24=&querytype_25=origem&query_relational_operator_25=contains&query_value_25=&querytype_26=unidade&query_relational_operator_26=contains&query_value_26=&querytype_27=status&query_relational_operator_27=contains&query_value_27=&querytype_28=curso&query_relational_operator_28=contains&query_value_28=&querytype_29=nivelDeEnsino&query_relational_operator_29=contains&query_value_29=&querytype_3=subject&query_relational_operator_3=contains&query_value_3=&querytype_30=tipoDeMaterial&query_relational_operator_30=contains&query_value_30=&querytype_4=dateIssued&query_relational_operator_4=contains&query_value_4=&querytype_5=dataAno&query_relational_operator_5=equals&query_value_5=&querytype_6=nivelAcademico&query_relational_operator_6=equals&query_value_6=&querytype_7=tipo&query_relational_operator_7=equals&query_value_7=&querytype_8=idioma&query_relational_operator_8=equals&query_value_8=&querytype_9=formatoArquivo&query_relational_operator_9=equals&query_value_9=&query=](https://lume.ufrgs.br/handle/10183/1/discover?rpp=10&etal=0&query=leprosy+resist*&group_by=none&page=2&querytype_0=title&query_relational_operator_0=contains&query_value_0=&querytype_1=authortd&query_relational_operator_1=contains&query_value_1=&querytype_10=serie&query_relational_operator_10=contains&query_value_10=&querytype_11=author&query_relational_operator_11=contains&query_value_11=&querytype_12=acervo&query_relational_operator_12=contains&query_value_12=&querytype_13=descriptionSection&query_relational_operator_13=contains&query_value_13=&querytype_14=tipoAto&query_relational_operator_14=contains&query_value_14=&querytype_15=natureza&query_relational_operator_15=contains&query_value_15=&querytype_16=numeroAto&query_relational_operator_16=contains&query_value_16=&querytype_17=orgao&query_relational_operator_17=contains&query_value_17=&querytype_18=dataFinal&query_relational_operator_18=contains&query_value_18=&querytype_19=programa&query_relational_operator_19=contains&query_value_19=&querytype_2=orientador&query_relational_operator_2=contains&query_value_2=&querytype_20=entrevistado&query_relational_operator_20=contains&query_value_20=&querytype_21=grandeArea&query_relational_operator_21=contains&query_value_21=&querytype_22=tipoDeApresentacao&query_relational_operator_22=contains&query_value_22=&querytype_23=areaTematica&query_relational_operator_23=contains&query_value_23=&querytype_24=coordenador&query_relational_operator_24=contains&query_value_24=&querytype_25=origem&query_relational_operator_25=contains&query_value_25=&querytype_26=unidade&query_relational_operator_26=contains&query_value_26=&querytype_27=status&query_relational_operator_27=contains&query_value_27=&querytype_28=curso&query_relational_operator_28=contains&query_value_28=&querytype_29=nivelDeEnsino&query_relational_operator_29=contains&query_value_29=&querytype_3=subject&query_relational_operator_3=contains&query_value_3=&querytype_30=tipoDeMaterial&query_relational_operator_30=contains&query_value_30=&querytype_4=dateIssued&query_relational_operator_4=contains&query_va)

81) Universidade Católica de Pelotas

<https://pos.ucpel.edu.br/ppgsc/?s=leprosy>

<https://pos.ucpel.edu.br/ppgsc/?s=leprosy+resist*>

82) Pontifícia Universidade Católica do Rio Grande do Sul

<http://tede2.pucrs.br/tede2/simple-search?query=leprosy>

<http://tede2.pucrs.br/tede2/simple-search?location=%2F&query=leprosy+resist*&rpp=10&sort_by=score&order=desc>

83) Universidade de Passo Fundo

<https://secure.upf.br/pergamum/biblioteca/index.php>

84) Universidade de Caxias do Sul

<https://repositorio.ucs.br/xmlui/handle/11338/37/discover>

<https://repositorio.ucs.br/xmlui/handle/11338/37/discover?query=leprosy+resist*&submit=>

85) Universidade do Vale do Rio dos Sinos

<http://www.repositorio.jesuita.org.br/handle/UNISINOS/1565/discover>

86) Universidade Luterana do Brasil

<http://www.ppgecim.ulbra.br/teses/index.php/ppgecim/search/search>

87) Universidade Federal de Rondônia

<http://ri.unir.br/jspui/simple-search?location=%2F&query=hansen%C3%ADase&rpp=10&sort_by=score&order=desc&filter_field_1=subject&filter_type_1=equals&filter_value_1=Hansen%C3%ADase>

<http://ri.unir.br/jspui/simple-search?location=%2F&query=hansen%C3%ADase+resist%C3%AAncia&rpp=10&sort_by=score&order=desc&filter_field_1=subject&filter_type_1=equals&filter_value_1=Hansen%C3%ADase>

88) Universidade Federal de Santa Catarina

<https://repositorio.ufsc.br/handle/123456789/74645/discover?query=leprosy+&submit=Ir&rpp=10>

<https://repositorio.ufsc.br/handle/123456789/74645/discover?query=leprosy+resist*+&submit=Ir&rpp=10>

89) Universidade do Estado de Santa Catarina

<http://www.tede.udesc.br/simple-search?query=leprosy>

<http://www.tede.udesc.br/simple-search?location=%2F&query=leprosy+resist*&rpp=10&sort_by=score&order=desc>

90) Universidade Regional de Blumenau

<https://bu.furb.br/consulta/novaConsulta/pesqPosGrad.php?acao=pesquisar&rdbtTpConsulta=10&ExpBusca=Leprosy&anoDefesaIni=--Todos+os+anos--&anoDefesaFim=--Todos+os+anos--&progPG=--Todos+os+programas--&Submit=Pesquisar>

91) Universidade Federal de Sergipe

<https://ri.ufs.br/handle/riufs/2145/browse?type=subject&order=ASC&rpp=20&value=Leprosy>

<https://ri.ufs.br/handle/riufs/2145/browse?type=subject&sort_by=1&order=ASC&rpp=20&etal=-1&value=Leprosy&starts_with=leprosy+resist*>
