# Supplementary material for: The efficacy of topical, oral and surgical interventions for the treatment of tungiasis: A systematic review of the literature
Source: PLoS Negl Trop Dis. 2021 Aug 20;15(8):e0009722. doi: 10.1371/journal.pntd.0009722 (PMC8409605; doi:10.1371/journal.pntd.0009722)
Supplement: Attachment — Submitted filename: Responses to reviewer comments.docx [file pntd.0009722.s005.docx]

Dear PLOS NTD Editors and Reviewers:

First, thank you very much for the fast and detailed review of the present manuscript. We are very grateful for all of the compliments registered by the reviewers, pointing to the importance and to the well-conducted methodology of our systematic review. We have agreed to and implemented all of the changes requested by reviewers #2 and #3, and we thank reviewer #1 for the acceptance recommendation. Please find below detailed responses to all of the reviewer requests, along with indications of text alterations.

Sincerely,

Ciro Martins Gomes, MD, PhD, Hospital Universitário de Brasília SGAN 605, Av. L2 Norte, CEP: 70910-900, Brasília, Distrito Federal, Brazil; Phone: [+]55 (61)9155-4039; Fax: [+]55 (61)3225-6799; Email: cirogomes@unb.br

**ANSWERS TO REVIEWERS’ COMMENTS:**

**Key Review Criteria Required for Acceptance?**

**Reviewer #1: (No Response)**

**Reviewer #2: The authors aimed to assess the efficacy of currently available topical, oral, and surgical interventions for the treatment of tungiasis, which is clearly delineated by the hypothesis. The study design is appropriate, with enough articles included for a PRISMA-compliant systematic review. Furthermore, the authors followed all applicable regulatory guidelines during this study.**

Thank you very much for the compliments.

**Reviewer #3: The purpose of this paper was to collect information and find the most efficacious treatment for Tungiasis, and this paper accomplishes that very well.**

Thank you very much for the compliments.

**Results**

**Reviewer #1: (No Response)**

**Reviewer #2: The presented results appear to be more about methods than key findings and should be updated. Furthermore, the information in the tables is detailed and easy to understand. The figures are adequate and of sufficient quality in accordance with the journal's guidelines.**

As requested, we have indicated, at the end of the introduction section, that the present review also aimed to analyse the current methodology used for the most relevant studies in the field (page 6 lines 128-134). We have also included detailed key findings of the most important studies in the tables, including the number of cure events in each study (Tables 2 and 3).

**Reviewer #3: The results and analysis were clear and the treatment overview was extremely helpful.**

Thank you very much for the compliments.

**Conclusions**

**Reviewer #1: (No Response)**

**Reviewer #2: Significant findings of this study support conclusion. This study's limitations are appropriately described. More robust discussion about the study's potential prospects for advancing public health relevance is required.**

As required, we have included a detailed statement indicating potential prospects for better treatment and prevention in tungiasis-endemic areas. Based on the scientific background and state-of-the-art methodological procedures, we have also recommended possible directions for future clinical trials, especially the need for environmental interventions for recurrence prevention (Page 11, lines 182 and 183; Page 23, lines 347-350).

**Reviewer #3: The conclusions are directly supported by the data. Limitations were explained and presented accordingly. The support of Dimeticone as treatment is well founded but in this paper there is a lack of explanation. The occlusive action is mentioned but not explained. I would include a section explaining the method of action of this treatment. I would also suggest that the authors include a image of the the different types of treatments after application, and if possible a graphic depicting what a individual Tunga penetrans looks like and why the occlusive nature of this treatment works.**

As requested, a detailed explanation related to the physical mechanism of action has been included. Since our study did not use clinical evaluation or patient follow-up, we included in the discussion section the requested explanations related to the dimeticone effect in different evolutionary phases of *T. penetrans* infection (Page 22, lines 320-328).

**Editorial and Data Presentation Modifications?**

**Reviewer #1: (No Response)**

**Reviewer #2: (No Response)**

**Reviewer #3: The paper does need some minor grammatical revisions for more concise wording and make the paper easier to read.**

As required, we have submitted the reviewed version of our article to a professional editing service. Significant improvements were made, and the text is now more didactic.

**Summary and General Comment**

**Reviewer #1: Manuscript PNTD-D-21-00651 reviewed the progress of treatment of the neglected disease tungiasis mostly occurred in South America and Africa. The clinical trials included in this review article supported the application of topical drugs such as dimeticones as the encouraging yet cost-effective therapy for the treatment of this disease. The paper is well organized and written. The conclusions drawn from this review paper are of great values for the clinical trials as well as the regulatory purposes in the affected countries. I recommend to accept this manuscript without further changes.**

Thank you very much for the acceptance recommendation.

**Reviewer #2: This is a well-executed important study with apparent strength. The review is registered prospectively, and the authors investigate the efficacy of various interventions for the treatment of tungiasis. To avoid publication and language bias, the review employs the Cochrane Risk of Bias Tool and the Critical Appraisal Tool. This article's rationale and novelty statement should be elaborated at the appropriate place. Here are the key issues with this article: What about the studies that were excluded during the qualitative analysis of the articles (case series, case reports, and trials)? How did they fail to contribute to the study, and how did their exclusion affect the study's outcome? The materials and methods must explicitly state how the studies' risk of bias and assessment of quality studies was judged. Please ensure that the search and inclusion of studies is as up to date as possible.**

As requested and as suggested in the PRISMA statement, we have included a novelty statement in the introduction section explaining that research in the field of ectoparasitosis is scarce (Page 6, lines 128-130). We have also included details related to the exclusion criteria and quality analysis. Following our registered protocol, we have included a statement related to excluded studies explaining that, since those studies used descriptive data, any effect related to the prescribed treatment would have suffered from serious bias (Page 10, lines 152-155). We aimed to include more reliable analytical studies (cohort studies and clinical trials) to promote better external validation and to better guide future efforts. Following systematic review of the literature guidelines, we have assured that the present review is up-to-date.

**Reviewer #3: Overall this paper adequately accomplishes its goal. I do want to reiterate the need for a explanation of the method of action of this treatment. Understanding how this treatment works on a physical level when compared to other treatments is of paramount importance.**

As requested, a detailed explanation related to dimeticone physical mechanism of action of has been included. Since our study did not use clinical evaluation or patient follow-up, we also included detailed explanations of this effect in different evolutionary phases of *T. penetrans* infection (Page 22, lines 320-328).
